# CFTR Modulators: Shedding Light on Precision Medicine for Cystic Fibrosis

**DOI:** 10.3389/fphar.2016.00275

**Published:** 2016-09-05

**Authors:** Miquéias Lopes-Pacheco

**Affiliations:** Institute of Biophysics Carlos Chagas Filho, Federal University of Rio de JaneiroRio de Janeiro, Brazil

**Keywords:** CFTR, cystic fibrosis, protein misfolding, intracellular trafficking, proteostasis network, personalized medicine, ABC transporters

## Abstract

Cystic fibrosis (CF) is the most common life-threatening monogenic disease aﬄicting Caucasian people. It affects the respiratory, gastrointestinal, glandular and reproductive systems. The major cause of morbidity and mortality in CF is the respiratory disorder caused by a vicious cycle of obstruction of the airways, inflammation and infection that leads to epithelial damage, tissue remodeling and end-stage lung disease. Over the past decades, life expectancy of CF patients has increased due to early diagnosis and improved treatments; however, these patients still present limited quality of life. Many attempts have been made to rescue CF transmembrane conductance regulator (CFTR) expression, function and stability, thereby overcoming the molecular basis of CF. Gene and protein variances caused by CFTR mutants lead to different CF phenotypes, which then require different treatments to quell the patients’ debilitating symptoms. In order to seek better approaches to treat CF patients and maximize therapeutic effects, CFTR mutants have been stratified into six groups (although several of these mutations present pleiotropic defects). The research with CFTR modulators (read-through agents, correctors, potentiators, stabilizers and amplifiers) has achieved remarkable progress, and these drugs are translating into pharmaceuticals and personalized treatments for CF patients. This review summarizes the main molecular and clinical features of CF, emphasizes the latest clinical trials using CFTR modulators, sheds light on the molecular mechanisms underlying these new and emerging treatments, and discusses the major breakthroughs and challenges to treating all CF patients.

## Introduction

**FIGURE 1:**
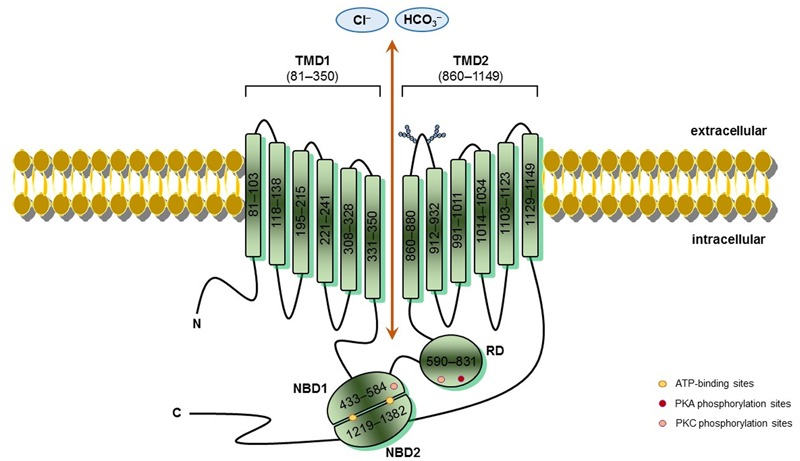
**CFTR schematic structure** – Cystic fibrosis transmembrane conductance regulator (CFTR) is a 1,480-amino acids protein inserted into the cell surface. CFTR possesses five domains: two transmembrane domains (TMD1/2), containing six hydrophobic alpha-helices, which cross the cell surface lipid bilayer, and are joined by two intracellular loops and three extracellular loops, and with glycosylated residues linked in the extracellular loop 4 (N894, N900); two nucleotide-binding domains (NBD1/2) with highly conserved sequenced for ATP-binding, where occur hydrolysis; and one regulatory domain (RD) with multiple phosphorylation sites. CFTR channel open when protein kinase A (PKA) and protein kinase C (PKC) phosphorylate RD and ATPs bind to side chain charged amino acids in NBDs, thereby activating CFTR function. TMDs form the gate where occurs chloride conductance. The positions denoted into the boxes correspond to the first and last amino acid of each fragment and CFTR sequence was obtained in the Cystic Fibrosis Mutation Database (CFTR1 database; http://www.genet.sickkids.on.ca/Home.html).

Mutations in the cystic fibrosis transmembrane conductance regulator (CFTR) gene cause cystic fibrosis (CF) – the most common life-limiting autosomal recessive inherited disorder in Caucasian people. The mutated gene translates into a defective CFTR protein with loss-of-activity (Kerem et al., 1989; Riordan et al., 1989). CFTR encodes an anion channel expressed in several cell types that: (1) transports chloride across the apical membrane; (2) modulates the activity of other ion channels, thereby regulating fluid and electrolyte balance in the mucosal membranes; and (3) secretes bicarbonate, which is crucial for pH regulation, host defense and protection against noxious stimuli (Gadsby et al., 2006; Gentzsch et al., 2010; Gustafsson et al., 2012). As a protein of the ATP-binding cassette (ABC) family, CFTR is comprised of two transmembrane domains (TMDs), two nucleotide-binding domains (NBDs) and a unique regulatory domain (RD) (Gadsby et al., 2006) (**Figure 1**). Cyclic AMP (cAMP)-dependent protein kinase A (PKA), protein kinase C (PKC) and ATP control its activity (Winter and Welsh, 1997; Chappe et al., 2003). The most prevalent CFTR mutation was discovered almost 30 years ago; it was the deletion of a phenylalanine at position 508 (ΔF508) (Kerem et al., 1989; Riordan et al., 1989), which affects ∼80% of CF patients worldwide (Bobadilla et al., 2002; Sosnay et al., 2013). The mutation ΔF508 reduces thermal and kinetic stability of NBD1 and precludes interdomain interactions (Serohijos et al., 2008; Mendoza et al., 2012). Hence, the endoplasmic reticulum (ER) retains misfolded ΔF508-CFTR, which forms only a partially glycosylated protein, and the proteasome promptly degrades it (Cheng et al., 1990; Jensen et al., 1995).

**FIGURE 2:**
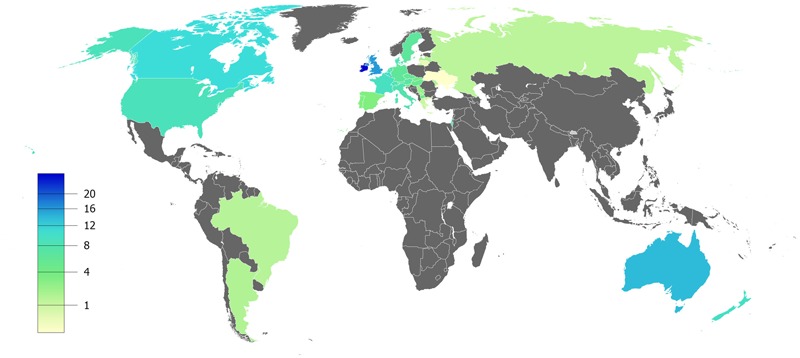
**Estimated prevalence of cystic fibrosis per 100,000 habitants** – Data compiled from the latest registry reports of Europe (European Cystic Fibrosis Society [ECFS], 2016), United States (Cystic Fibrosis Foundation [CFF], 2015), Canada (Cystic Fibrosis Canada [CFC], 2016), Australia (Cystic Fibrosis Federation Australia [CFFA], 2016) and Brazil (Brazilian Cystic Fibrosis Study Group [GBEFC], 2016).

**FIGURE 3:**
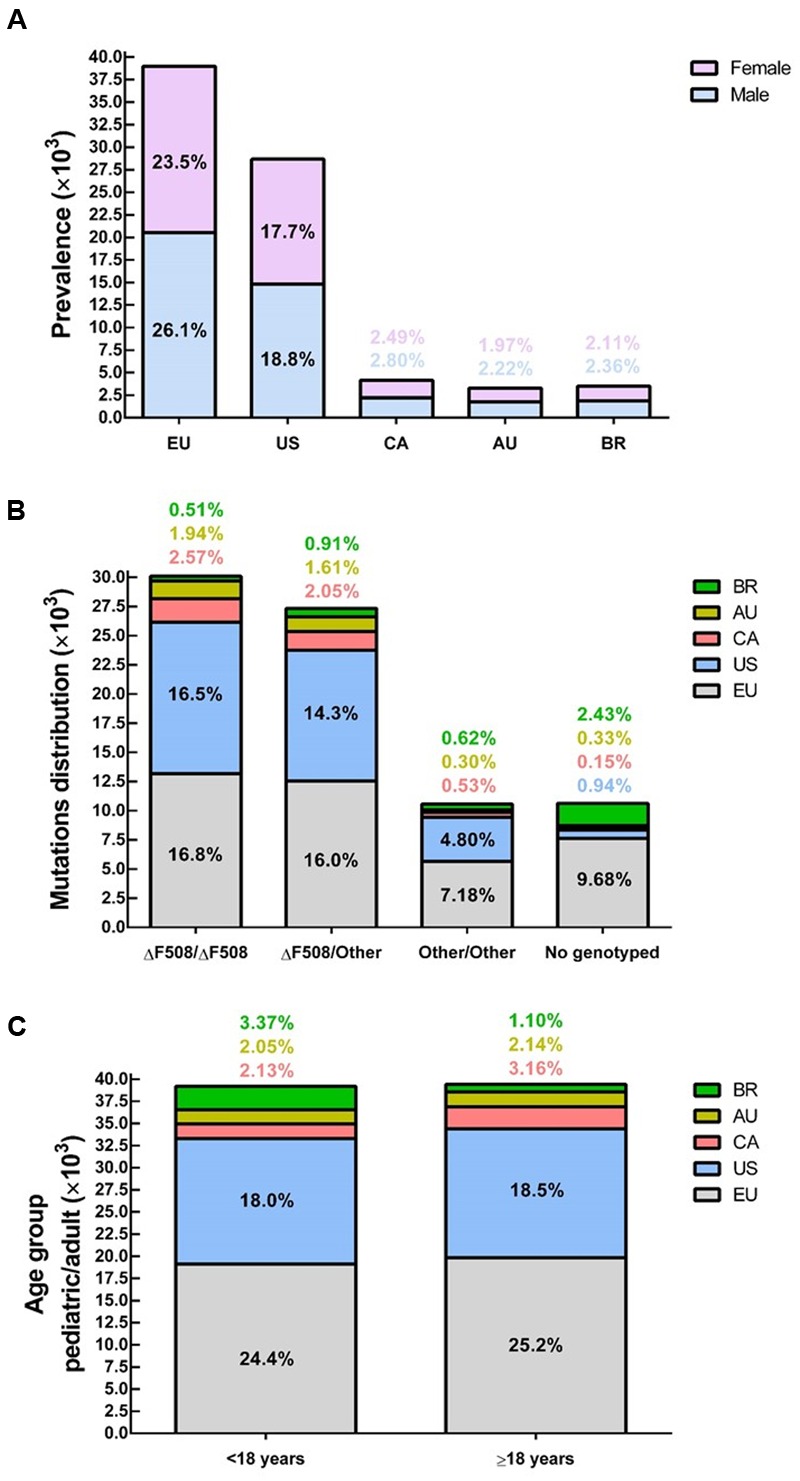
**Demography of cystic fibrosis in a sample of 78,627 patients in different countries or demographic regions** – **(A)** Prevalence by gender: average of 52% male and 48% female. **(B)** Within the sample group, 86% have been genotyped and approximately 38% are ΔF508-homozygous, 35% ΔF508-heterozygous and 13% bearing other CFTR (cystic fibrosis transmembrane conductance regulator) mutants in both alleles. **(C)** About 50% of patients are under 18 years and 50% are 18 years or older. Data compiled from the latest registry reports of Europe (EU; European Cystic Fibrosis Society [ECFS], 2016), United States (US; Cystic Fibrosis Foundation [CFF], 2015), Canada (CA; Cystic Fibrosis Canada [CFC], 2016), Australia (AU; Cystic Fibrosis Federation Australia [CFFA], 2016) and Brazil (BR; Brazilian Cystic Fibrosis Study Group [GBEFC], 2016).

**FIGURE 4:**
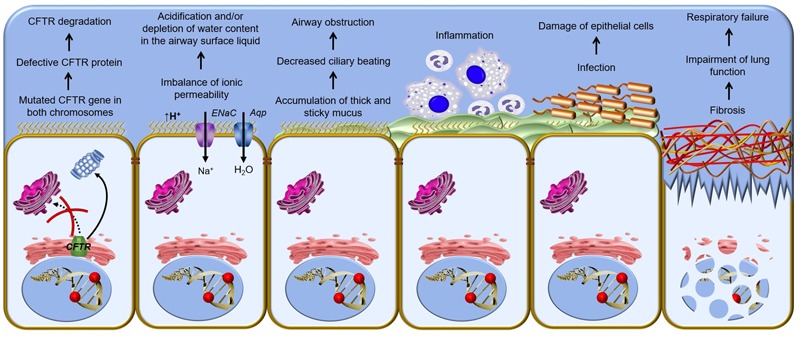
**Pathophysiological cascade of respiratory disorder in cystic fibrosis** – Cellular mechanism of cystic fibrosis begins with the defective CFTR (cystic fibrosis transmembrane conductance regulator) gene and shortage of CFTR channel at the plasma membrane. A vicious cycle of airways obstruction, inflammation and infection leads to epithelial damage, lung remodeling and end-stage lung disease. ENaC, epithelial Na^+^ channel; Aqp, aquaporin.

CF is a systemic disease that affects ∼85,000 people and presents heterogeneous distribution globally (Bobadilla et al., 2002; Sosnay et al., 2013) (**Figure 2**, **Table 1**). According to the latest registry reports, 38,985 CF patients are in Europe (European Cystic Fibrosis Society [ECFS], 2016), 28,676 in the United States (Cystic Fibrosis Foundation [CFF], 2015), 4,128 in Canada (Cystic Fibrosis Canada [CFC], 2016), 3,294 in Australia (Cystic Fibrosis Federation Australia [CFFA], 2016) and 3,511 in Brazil (Brazilian Cystic Fibrosis Study Group [GBEFC], 2016) (**Figure 3**). The major cause of morbidity and mortality in CF is the respiratory disorder caused by the lack of CFTR at the plasma membrane (PM) (Moskowitz et al., 2008), which decreases the anion permeability in airway cells and leads to a progressive pathophysiological cascade (**Figure 4**): (1) A faulty conductance of other ions, such as excessive sodium transport mediated by epithelial Na^+^ channel (ENaC), since it is negatively regulated by CFTR (Kunzelmann et al., 1995; Gentzsch et al., 2010). Controversially, some reports have shown that CFTR loss-of-activity reduces chloride conductance without increasing sodium absorption in CF epithelia (Chen et al., 2010; Itani et al., 2011). (2) The imbalance of ion regulation depletes water content and/or decreases pH in the airway surface liquid (Tarran et al., 2005; Tang et al., 2016). (3) Dehydration and/or acidification of epithelial lining, as well as increased mucin polymer cross-links, raise the amount and viscosity of mucus in gel phase (Tarran et al., 2005; Yuan et al., 2015; Shah et al., 2016; Tang et al., 2016), making the mucus tenacious and difficult to remove by ciliary beating. (4) Accumulation of mucus leads to obstruction of the airways, inflammation and bronchiectasis. (5) Frequently, pathogens colonize the airways and increase the recruitment of inflammatory cells (Lyczak et al., 2002; Bhatt, 2013). Furthermore, oxygen depletion below the sputum-air interface favors biofilm formation (Worlitzsch et al., 2002; Cowley et al., 2015). (6) The destruction of airway and lung parenchyma epithelial cells causes tissue remodeling, reduction of gas exchange area and impairment of lung function. (7) As the disease progresses, the patient succumbs to death due to respiratory failure.

**Table 1:** Top 10 countries with the highest number of CF patients.

	Registered patients	Per 100,000 habitants
1°	United States	Ireland
2°	United Kingdom	United Kingdom
3°	France	Australia
4°	Germany	Canada
5°	Italy	Belgium
6°	Canada	New Zealand
7°	Brazil	France
8°	Australia	United States
9°	Russia	Switzerland
10°	Spain	Denmark

In addition to the substantial impact of CFTR dysfunction in the upper and lower respiratory tract, CF patients also experience clinical manifestations related to the reproductive, gastrointestinal and glandular systems (**Table 2**). Male infertility is present in 98% of cases, and about 85% of CF patients have pancreatic insufficiency, often associated to malnutrition.

**Table 2:** Approximate age of onset of CF clinical manifestations and comorbidities.

	Upper and lower respiratory tract	Gastrointestinal and hepatobiliary systems	Endocrine and reproductive systems	Salt-wasting syndrome and others
Babyhood and childhood	Chronic cough Sputum overproduction Tenacious mucus Airway obstruction Recurrent and persistent pneumonia or lung infections Bronchiectasis Nasal polyps/sinus disease	Meconium ileus Steatorrhea Deficiency of fat-soluble vitamins Intussusception Recurrent pancreatitis Pancreatic insufficiency	Absence of the vas deferens Impaired growth	Salty sweat Mucosal dehydration Hypochloremia Hyponatremia Hypokalemia Digital clubbing
Adolescence and adulthood	Atelectasis Impaired pulmonary function Hemoptysis Chronic infection with multidrug-resistant pathogens Allergic bronchopulmonary aspergillosis Pneumothorax Respiratory failure	Biliary fibrosis/cirrhosis Hepatic steatosis Gastroesophageal reflux Distal intestinal obstruction syndrome Digestive tract cancer	Delayed puberty Oligomenorrhea Reduced fertility in women Obstructive azoospermia in males CF-related *diabetes mellitus* Reduced bone mineral density/osteoporosis	Arthritis/vasculitis Nephrolithiasis Chronic kidney disease Anxiety/depression

*Please note that the clinical signs and symptoms in an approximate age of onset do not exclude the possibility of these arose from early or late manner.*

Salt loss syndrome also affects CF patients, since chloride reabsorption via CFTR in the sweat glands is compromised, increasing chloride elimination (>60 mmol.L^-1^), as well as of other ions. In fact, the iontophoretic sweat test is considered the ‘gold standard’ for the diagnosis of CF. It precedes only the confirmation of a diagnosis by genetic testing (Lyczak et al., 2002; Farrell et al., 2008; Castellani et al., 2016). In addition, sweat chloride measurement is a biomarker of CFTR activity and response for new treatments (Accurso et al., 2014; Collaco et al., 2016).

## Life Expectancy in Cystic Fibrosis

**FIGURE 5:**
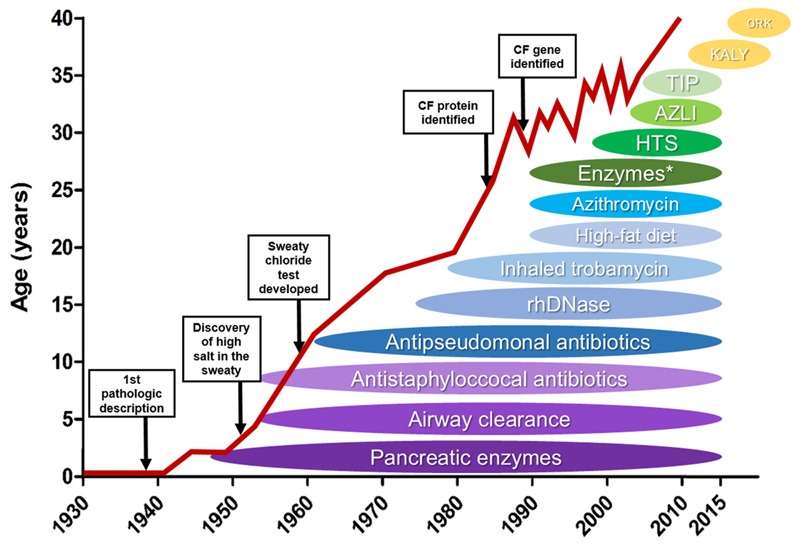
**Effect of novel therapies on life expectancy of cystic fibrosis patients** – Schematic illustration of how the discovery and introduction of novel cystic fibrosis (CF) treatments have influenced the patients’ survival over the decades. HTS: high throughput screening, AZLI, aztreonam for inhalation solution; TIP, tobramycin inhalation solution; KALY, Kalydeco^TM^; ORK, Orkambi^TM^. ^∗^enteric-coated pancreatic enzymes. (Reproduced and adapted with permission of European Respiratory Society©: *The European Lung White Book Respiratory Health and Disease in Europe*, 2^nd^ Ed. © 2013 European Respiratory Society, Sheffield, UK. Print ISBN: 978-1-84984-042-2, Online ISBN: 978-1-84984-043-9).

Multifactorial improvements have increased the survival of CF patients (**Figure 5**): early diagnosis, genotype–phenotype detection, nutritional support, more efficient and effective pulmonary interventions, multidisciplinary professional monitoring, establishment of CF specialist centers and, most recently, the development of precision medicine (Farrell et al., 2008; Cohen-Cymberknoh et al., 2011; Smyth et al., 2014; Quon and Rowe, 2016).

In the last decades, many countries have adopted newborn screening programs. Newborns are diagnosed through the measurement of immunoreactive trypsinogen. This early diagnosis and the confirmation of the disease through the sweat test are important for a better prognosis (Farrell et al., 2008; Djik and Fitzgerald, 2012; Castellani et al., 2016). Thereafter, identifying which are the mutations in the CFTR gene is crucial, since different ethnic or regional populations can have a different spectrum of CFTR variants, each leading to different degrees of disease severity (Moskowitz et al., 2008; Pique et al., 2016; Schrijver et al., 2016).

A multidisciplinary team of health professionals should perform periodic monitoring of the disease progression and make any necessary adjustments in treatments in order for patients to achieve the best clinical outcomes (Cohen-Cymberknoh et al., 2011; Smyth et al., 2014). CF patients show weight loss and impaired growth due to pancreatic insufficiency and intestinal malabsorption. Supplementations with pancreatic enzymes and fat-soluble vitamins have improved their nutritional status (Feranchak et al., 1999; Kalnins and Wilschanski, 2012). Lung injuries are the most common clinical characteristic due to mucociliary clearance impairment in the airways. Production of very viscous mucus impairs the ciliary beating, and treatments with mucolytic (e.g., dornase-alpha) (Fuchs et al., 1994) or hydrator (e.g., mannitol, hypertonic saline) (Elkins et al., 2006; Bilton et al., 2011) are helpful in eliminating the retained mucus. Antibiotic therapy is also essential, since the mucus becomes trapped in the respiratory tract, causing recurrent and persistent infections by a group of opportunistic pathogens: *Burkholderia cepacia, Haemophilus influenzae, Pseudomonas aeruginosa* (hallmark of CF), *Staphylococcus aureus* and *Stenotrophomonas maltophilia* (Lyczak et al., 2002; Worlitzsch et al., 2002; Bonestroo et al., 2010; Bhatt, 2013). It is noteworthy that during infection by *P. aeruginosa*, the bacterial population can segregate and evolve differently according to the lung microenvironment, leading to differences in the bacterial characteristics, such as nutritional requirements, defense and microbial resistance. This may explain, partly, why this pathogen is so prevalent and difficult to eradicate from CF patient lungs (Jorth et al., 2015; LaFayette et al., 2015).

Non-pharmacological treatments, such as aerobic exercise, physiotherapy, feeding and physiological supports, are also important to high-quality care and better outcomes (Cohen-Cymberknoh et al., 2011; Smyth et al., 2014). As the disease progresses, patients require continuous therapy with oxygen, and in the end-stage lung disease, the only alternative is lung transplantation, which still presents a high risk of cellular rejection (Adler et al., 2009; Calabrese et al., 2015).

Over the past decades, all the above-mentioned improvements have lengthened the life expectancy of CF patients. In 1960, CF patients only lived through childhood. Nowaday, they live to see their forties (**Figure 5**), and those born more recently are expected to make it to their fifties (Cohen-Cymberknoh et al., 2011; MacKenzie et al., 2014; Burgel et al., 2015). However, CF patients still present even more reduced life expectancy – between 20 and 30 years – in some regions of the world, including Brazil and the African continent (Marson et al., 2015; Stewart and Pepper, 2016).

Several new complications associated with increased survival (**Table 2**), which were rare or not previously observed, pose new challenges for CF scholars. CF-related diabetes, metabolic bone disease and multidrug-resistant pulmonary pathogens are some comorbidities in older CF patients (Plant et al., 2013). Although still uncommon, adult CF patients have a higher risk of gastrointestinal cancer compared to an age-matched non-CF population (Maisonneuve et al., 2013), which may be explained by the fact that CFTR is a tumor suppressor gene (Than et al., 2016). Another severe complication is the allergic bronchopulmonary aspergillosis, which is correlated to accelerated decline of lung function in adult CF patients (Armstead et al., 2014).

Despite the increase in life expectancy, CF patients still present a limited quality of life due to the high costs and the burden of treatments needed. In particular, CF patients living in developing countries tend to have worse clinical outcomes compared to those who live in developed countries, since lower-income countries have scarce financial resources to optimize therapies, including the application of precision medicine (Cohen-Cymberknoh et al., 2016). Precision medicine (or personalized treatment) is a new approach that takes into account the genetic variances of each individual. Therefore, CF patients with different CFTR mutations may require different treatments to quell their debilitating symptoms (Quon and Rowe, 2016). The knowledge obtained about the molecular scenario in which CFTR mutants are degraded has contributed to understanding the different defects caused by different mutants, and thus to developing drugs that rectify the primary defect of specific mutations.

## A Proteostasis Network Engaged in CFTR Degradation

CFTR biogenesis is a cellular process that involves several steps: post-transcriptional splicing, protein translation, folding at the ER, glycosylation at the Golgi apparatus, trafficking to the apical membrane, endosomal recycling and retrieval. The cellular and transcellular protein trafficking involves multiple quality control systems to compensate the limited fidelity of each system. Therefore, a protein homeostasis (proteostasis) network carefully checks CFTR maturation pathway (Balch et al., 2011). Among the quality control systems, ubiquitin-proteasome pathway destroys the largest fraction of misfolded CFTR (Cheng et al., 1990; Jensen et al., 1995), and aggresomes degrade by autophagy CFTR molecules that proteasomes cannot degrade (Kawaguchi et al., 2003; Luciani et al., 2010). In addition, lysosomes eliminate non-native CFTR that escapes from ER-associated degradation (ERAD) (Sharma et al., 2004; Glozman et al., 2009; Okiyoneda et al., 2010). The mutant ΔF508 fails to achieve the native conformation and ERAD machinery arrests CFTR, thereby precluding the protein from being delivered to the PM (Cheng et al., 1990; Jensen et al., 1995).

**FIGURE 6:**
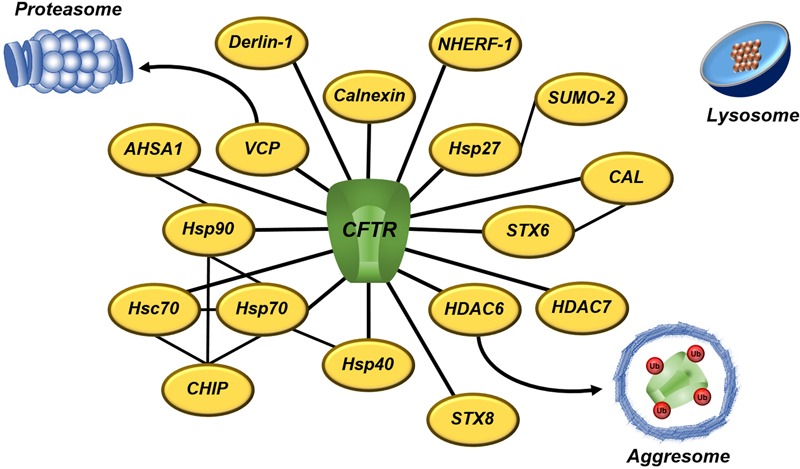
**A subset of proteostasis network engaged to CFTR degradation** – CFTR interactome involves several quality control proteins that directly or indirectly target CFTR to degradation. Proteasomes and aggresomes eliminate CFTR that fails in acquire the native conformation. Lysosomes degrade CFTR removed from the cell surface during the recycling. The black lines denoted the interaction between CFTR and proteostasis components. AHSA1, activator of 90 kDA Hsp ATPase homolog 1; CAL, CFTR-associated ligand; CHIP, carboxyl terminus of Hsc70-interacting protein; CFTR, cystic fibrosis transmembrane conductance regulator; HDAC, histone deacetylase; Hsc, heat-shock cognate; Hsp, heat-shock protein; NHERF, Na^+^/H^+^ exchanger regulatory factor; SUMO, small ubiquitin-like modifier; STX, syntaxin; Ub, ubiquitin; and VCP, vasolin-containing protein.

CFTR folding is facilitated by many molecular chaperones and co-chaperones, which form a CFTR interactome; however, unstable and misfolded CFTR remains bound to chaperones, promoting premature degradation of CFTR (Balch et al., 2011; Amaral and Farinha, 2013; Kim et al., 2013; Pankow et al., 2015) (**Figure 6**). The heat shock protein (Hsp)27 (or HspB1) binds ΔF508 with small ubiquitin-like modifier (SUMO)-2, directing it to be degraded via SUMOylation (Ahner et al., 2013; Gong et al., 2016). Hsp27 also prevents protein aggregation during stress (Ahner et al., 2007). Hsp40 (or DnaJ) sequesters misfolded CFTR for ERAD and it works as a co-chaperone for Hsp70 (Meacham et al., 1999; Farinha et al., 2002; Kakoi et al., 2013). Hsp70 is a core ER chaperone and its prolonged association with CFTR mutant results in CFTR ubiquitination and degradation by the 26S proteasome (Yang et al., 1993; Jensen et al., 1995; Meacham et al., 2001; Sun et al., 2006). Hsp70 connects to heat shock cognate (Hsc)70, forming the first ER checkpoint that retains the mutant ΔF508. Hsc70 couples to its co-chaperone, the carboxyl terminus of Hsc70-interacting protein (CHIP), and leads CFTR mutant to degradation (Meacham et al., 1999, 2001; Farinha and Amaral, 2005). Hsp90 helps wild type (wt)-CFTR to achieve the complete folding, but it also forms a ‘chaperones trap’ with Hsp40/Hsp70 that targets ΔF508 for proteasome degradation (Wang et al., 2006; Ahner et al., 2007; Koulov et al., 2010; Coppinger et al., 2012). The ATPase activity of Hsp90 is regulated by its co-chaperone, the activator of 90kDa Hsp APTase homolog 1 (AHSA1 or Aha1) (Wang et al., 2006; Koulov et al., 2010). Calnexin is an ER transmembrane chaperone that acts as second checkpoint, assessing CFTR folding status and sending the misfolded protein for degradation (Farinha and Amaral, 2005; Okiyoneda et al., 2008).

In addition to the molecular chaperones, inhibition of the histone deacetylase (HDAC) (Hutt et al., 2010; Pankow et al., 2015) or the vasolin-containing protein (VCP or p97) (Vij et al., 2006) rescues the trafficking and gating of ΔF508-CFTR. VCP (Boyault et al., 2006) and HDAC6 (Kawaguchi et al., 2003) translocate misfolded proteins to proteasomes and aggresomes, respectively. VCP is associated to ubiquitinated CFTR, whereas derlin-1 recognizes misfolded, non-ubiquitinated CFTR and initiates its dislocation and degradation in the early course of CFTR biogenesis (Sun et al., 2006). The CFTR-associated ligand (CAL) is a PDZ domain-containing protein, located primarily at Golgi apparatus, which modulates surface expression of CFTR (Cheng et al., 2002). CAL forms a complex with syntaxin (STX)6 that promotes CFTR degradation in lysosomes (Cheng et al., 2010; Cheng and Guggino, 2013). STX8 is an endosomal protein that impairs CFTR trafficking and inhibits its chloride channel activity by soluble *N*-ethylmaleimide-sensitive factor attachment protein receptor (SNARE) machinery (Bilan et al., 2004). Vasoactive intestinal peptide (VIP) is a neuropeptide that promotes interaction between CFTR and Na^+^/H^+^ exchanger regulatory factor 1 (NHERF-1) (Alshafie et al., 2014). NHERF-1 is a CFTR-binding protein that regulates CFTR distribution and function at the PM (Guerra et al., 2005; Kwon et al., 2007; Favia et al., 2010). Moreover, a system of peripheral proteins quality control (PPQC) removes CFTR from the PM if that is recognized as improperly folded (Okiyoneda et al., 2010).

In healthy conditions, the proteostasis network maintains a healthful proteome by integrating transcription, translation, folding and trafficking systems. However, a decline in the cellular proteostasis capacity occurs with aging, as well as protein misfolding/aggregation disorders cause an imbalance in the expression and/or binding of proteostasis components with client proteins, which accentuates the importance of a healthy quality control system (Balch et al., 2011; Kim et al., 2013; Villella et al., 2013). CFTR protein must fold into well-defined three-dimensional structure to attain stability and functionality (Balch et al., 2011; Kim et al., 2013). The protein lack or its incorrect folding prevents CFTR from performing its normal function and cells may respond to that by maintaining an activated heat shock response and leading to a maladaptive stress response. This effort to neutralize misfolding protein accumulation causes an additional stress to cells that may result in the exacerbation of disease symptoms. These findings were observed in cell lines and primary epithelium from CF and other misfolding protein diseases: alpha-1-antitrypsin, Alzheimer’s disease and Niemann-Pick type C1 disease (Roth et al., 2014). Another important determinant in inherited disorders is that the disease severity depends of the genetic background of each individual and not solely on the defect of a particular gene (Vu et al., 2015). CFTR gene is located at the chr7q31.2 and a genome-wide association analysis identified five loci that display significant relevance in the variable manifestation and progression of lung disease in CF (chr3q29, chr5p15.3, chr6p21.3, chr11p12-p13 and chrXq22-q23) (Corvol et al., 2015; Dang et al., 2016).

## Classes of CFTR Mutations and CFTR Modulators

**FIGURE 7:**
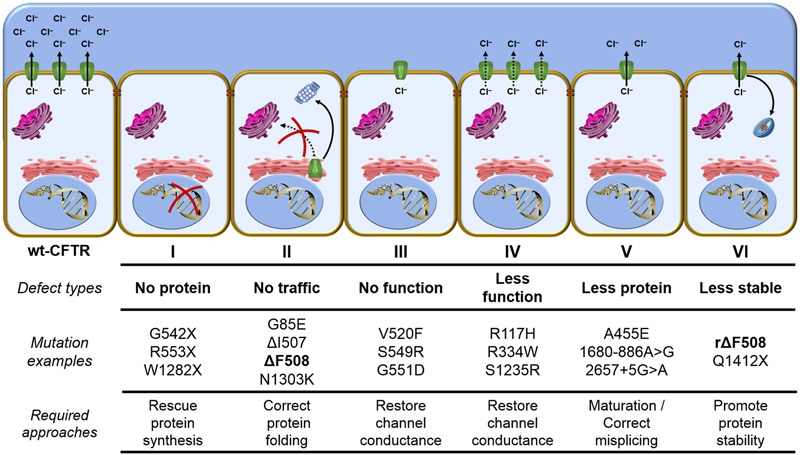
**Classes of CFTR mutations** – Distribution of CFTR mutations into six functional classes according to the primary molecular defect: *Class I mutants* are no protein synthesis, since the presence of premature stop codons (class Ia) or frameshifts for deletions or insertions (class Ib) preclude translation of full-length CFTR. *Class II mutants* are impaired trafficking protein, since CFTR fails to acquire complete folding and ER-associated degradation (ERAD) machinery eliminate the protein. *Class III mutants* are defective channel gating, since CFTR reach the cell surface, but it does not exhibit channel gating due to diminished ATP binding and hydrolysis. *Class IV mutants* are less functional proteins, since channel amount that achieve the plasma membrane could be similar to wt-CFTR, but it presents reduced chloride conductance. *Class V mutants* are less protein maturation caused by amino acid substitution or alternative splicing, since the protein amount that reaches the cell surface is reduced and it also leads to loss of chloride transport due to reduction in the quantity of CFTR channels. *Class VI mutants* are less stable protein, since CFTR at the plasma membrane is removed during the recycling and it is sent for lysosome degradation. wt, wild type; CFTR, cystic fibrosis transmembrane conductance regulator; rΔF508, rescued ΔF508 by low-temperature incubation; and ER, endoplasmic reticulum.

The ΔF508 is the most prevalent CFTR mutation with about 60% of CF patients’ chromosomes worldwide presenting this mutant (Bobadilla et al., 2002; Sosnay et al., 2013) (**Figure 3**). The remaining 40% present other CFTR mutations and there have been nearby 2,000 mutations reported in the Cystic Fibrosis Mutation Database (CFTR1 database)^1^. CFTR mutants can reduce protein expression, function, stability or a combination of these, and to help in understanding the nature of such gene and protein variability, CFTR defects have been classified into six classes (Amaral and Farinha, 2013; Quon and Rowe, 2016; Veit et al., 2016a) (**Figure 7**).

Distribution of CFTR mutants into classes may contribute to the application of precision medicine, since similar strategies might rescue CFTR from similar defects. However, the classification has a few caveats: (1) Numerous mutations have not been characterized, with respect to which group they should be allocated. The mutations’ characteristics for a subset of known mutations can be found at the Clinical and Functional Translation of CFTR (CFTR2 database)^2^. (2) At first glance, CFTR mutations in the same group show similar characteristics, but they may respond differently to the same treatment. (3) Several mutations (e.g., ΔF508) present pleiotropic defects, which means they could fit in more than one class. The major characteristic of ΔF508 is the incomplete folding of the protein caused by NBD1 instability (class II) (Cheng et al., 1990; Jensen et al., 1995; Lukacs and Verkman, 2012), but this mutation also affects channel gating (class III) (Dalemans et al., 1991; Serohijos et al., 2008; Mendoza et al., 2012) and cell surface residence time (class VI) (Sharma et al., 2004; Swiatecka-Urban et al., 2005; Okiyoneda et al., 2010). Based on this limitation, new classifications are under debate, including a scheme that would be composed of the traditional classes I, II, III/IV, V, VI and their 26 combinations, totaling 31 CFTR mutations classes (Veit et al., 2016a).

Monotherapies could be efficient in overcoming the molecular defect of some CFTR mutations; however, given the complexity of pleiotropic CFTR variants, combination of treatments may be required to rectify their defects and thus achieve therapeutic levels in the patients (Amaral and Farinha, 2013; Quon and Rowe, 2016; Veit et al., 2016a). Libraries of compounds have been screened by high-throughput screening (HTS) to identify more efficacious and non-cytotoxic drugs for application of precision medicine. Among these new pharmacological treatments, ‘CFTR modulators’ are small molecules that target specific defects caused by mutations in the CFTR gene and they are classified into five main groups: read-through agents, correctors, potentiators, stabilizers and amplifiers.

### Rescuing the Protein Synthesis

Read-through agents could benefit CF patients bearing class I mutations, since the presence of a premature stop codon (class Ia) precludes protein synthesis of full-length CFTR (Quon and Rowe, 2016). Class Ia mutations represent about 9% of the mutants causing CF and they are present in more than 50% of Israeli CF patients (Kerem et al., 1997; Bobadilla et al., 2002). In this line, ribosomal ‘over-reading’ of a premature stop codon should permit the continuing translation to the normal end of the transcript. This read-through effect was first observed with aminoglycoside antibiotics, such as gentamicin and tobramycin, which are commonly used for eradication of *P. aeruginosa*. Both antibiotics promoted expression of full-length CFTR at the PM and restored partially its chloride secretion in cell lines and transgenic mice (Howard et al., 1996; Du et al., 2002; Wilschanski et al., 2003). Despite these preclinical results, CF patients bearing nonsense mutations did not present CFTR activity after nasal application of aminoglycosides (Clancy et al., 2007). Furthermore, high systemic levels or long-term use of gentamycin has potential toxic effects in CF patients (Prayle et al., 2010).

Ataluren (formerly PTC124) is among the new drugs discovered by HTS. This drug suppressed the human G542X nonsense mutant in transgenic mice, restoring CFTR functional expression at the PM (Du et al., 2008). Thereafter, this drug resulted in some improvement in forced expiratory volume in 1 s (FEV_1_) of CF patients bearing nonsense mutations in phase II trials (Kerem et al., 2008; Sermet-Gaudelus et al., 2010; Wilschanski et al., 2011). However, these findings were inconclusive in the first long-term phase III trial, with only a subgroup of patients who were not receiving chronic inhaled aminoglycosides presenting a slight effect in FEV_1_ (Kerem et al., 2014). Ataluren has also shown limited premature stop codon suppression in CF rectal organoids (Zomer-van Ommen et al., 2016). Moreover, other read-through agents have shown promising results in preclinical experiments, including synthetic aminoglycosides (Rowe et al., 2011; Xue et al., 2014), ataluren derivatives (Pibiri et al., 2015, 2016), and the US Food and Drug Administration (FDA)-approved herbal agent, escin (Mutyam et al., 2016).

For other class I mutations (class Ib), such as frameshifts caused by small deletions or insertions during protein synthesis, very little has been achieved in treatments yet.

### Rescuing the Protein Folding and Trafficking

Correctors are small-molecules that enhance the conformational stability of CFTR, resulting in greater efficacy of protein folding and rescuing the trafficking of the mature CFTR to the PM (Pedemonte et al., 2005; Okiyoneda et al., 2013; Lopes-Pacheco et al., 2015; Quon and Rowe, 2016). CF patients bearing class II mutations, including ΔF508, could benefit from correctors treatment, since these CFTR mutants fail to reach complete folding and the ER machinery targets the protein to be degraded (Van Goor et al., 2006; Atawade et al., 2015; Rapino et al., 2015; Lopes-Pacheco et al., 2016).

New treatments may target the defective CFTR structure directly by binding to the mutated protein (pharmacological chaperone) and/or indirectly by modulating CFTR interactome (proteostasis regulator). Some reports have shown that correctors act either as pharmacological chaperones (Wang et al., 2007a; Sampson et al., 2011; Eckford et al., 2014; Sinha et al., 2015) or as proteostasis regulators (Hegde et al., 2015; Lopes-Pacheco et al., 2015, 2016; Rapino et al., 2015). Based on the possible mechanism of action as pharmacological chaperones, correctors have been classified into three groups: 1) correctors that stabilize the interactions between NBD1 and intracellular loops 1 and 4 (e.g., C3, C18 and VX-809); 2) correctors that restore NBD2 stability and its interfaces with other CFTR domains (e.g., C4); and 3) correctors that directly stabilize NBD1 (Okiyoneda et al., 2013).

Lumacaftor (formerly VX-809) restored ΔF508-CFTR expression and function in human bronchial epithelial (HBE) cells (Van Goor et al., 2011). The higher efficacy of lumacaftor, compared to other correctors (C3 and C4), seems to be due to its effect on the early CFTR synthesis (Farinha et al., 2015). Despite progress *in vitro*, lumacaftor treatment by itself showed a significant decrease only in sweat chloride levels and no improvements in FEV_1_ in a phase II trial with ΔF508-homozygous patients (Clancy et al., 2012). Lumacaftor presented the most variable effects in primary HBE cells and rectal organoids bearing ΔF508 in only one allele or other CFTR mutants in both alleles, with some mutants being ‘un-rescuable’ (e.g., N1303K) (Atawade et al., 2015; Dekkers et al., 2016a,b). Furthermore, a synonymous mutation changing ATC to ATT at position 507 (I507) alters mRNA and protein structure (Lazrak et al., 2013), consequently, affecting the efficacy of correctors to rescue ΔF508-CFTR (Bali et al., 2016a,b). Aiming at greater effects, combinations of correctors have been evaluated and have been shown to enhance the rescue of CFTR bearing ΔF508, as well as other class II mutants, compared to monotherapies (Okiyoneda et al., 2013; Phuan et al., 2014; Hegde et al., 2015; Lopes-Pacheco et al., 2015, 2016; Rapino et al., 2015). Some correctors could also increase CFTR maturation of class V mutants caused by amino acid substitution, such as A455E (Dekkers et al., 2016a,b; Lopes-Pacheco et al., 2016). In addition, VIP rescued functional expression of ΔF508-CFTR by stimulating both PKA- and PKC-dependent pathways in nasal and bronchial epithelial cells (Rafferty et al., 2009; Alcolado et al., 2011). Many newly discovered correctors are being investigated, including VX-661 that presented more a favorable pharmacokinetic profile than lumacaftor (Cholon et al., 2014; Veit et al., 2014; Phuan et al., 2015) and is in phase III trials to ΔF508-homozygous and -heterozygous patients (NCT02347657, NCT02392234 and NCT02412111).

Besides the correctors, drugs that modulate proteostasis have been evaluated to restore CFTR functional expression at the PM, since wt-CFTR and ΔF508 present a rather different interactome during their processing and trafficking (Pankow et al., 2015). Cysteamine, a proteostasis regulator approved by the FDA for nephropathic cystinosis, has shown promise for treating CF. Cysteamine (reduced form of cystamine), in association with epigallocatechin gallate, restored beclin 1-dependent autophagy protein levels and depleted sequestrosome 1/p62, thereby correcting autophagy flux, and rescuing CFTR trafficking, function and stability at the PM. These findings were observed in lungs from Cftr^ΔF508^ mice, CFBE41o- cell line expressing ΔF508-CFTR, and primary nasal epithelial cells freshly harvested from ΔF508-homozygous patients (Luciani et al., 2012; Villella et al., 2013; De Stefano et al., 2014). Recently, a combination of cysteamine and epigallocatechin gallate decreased sweat chloride levels, as well as inflammatory biomarkers levels in the sputum, and tended to improve FEV_1_ in ΔF508-homozygous and -heterozygous (with a class II mutant in the second allele) patients in a phase II trial (Tosco et al., 2016). Several other proteostasis regulators have been investigated to rescue CFTR, including sildenafil analogs (Robert et al., 2008), oubain (Zhang et al., 2012), roscovitine (Norez et al., 2014), suberoylanilide hydroxamic acid (Hutt et al., 2010; Pankow et al., 2015) and latonduine analogs (Carlile et al., 2016). Silencing of RPL12, a ribosomal stalk protein, also rescued ΔF508-CFTR and presented a synergistic effect with lumacaftor, restoring the mutant function to about 50% of the wt-CFTR in primary HBE cells bearing ΔF508 in both alleles (Veit et al., 2016b).

### Restoring the Channel Conductance

Potentiators are drugs that increase channel open probability, improving CFTR channel activity. Potentiators could benefit CF patients bearing class III and IV mutations, since CFTR is present at the PM, but it exhibits no gating or reduced activity (Van Goor et al., 2006, 2009, 2014; Eckford et al., 2012). Furthermore, patients bearing class I or II mutations for which protein synthesis or trafficking were rescued, but not proper channel activity, may benefit from this additional approach.

Ivacaftor (formerly VX-770) rescued CFTR channel gating in HBE cells bearing G551D mutation (Van Goor et al., 2009) through a nonconventional ATP-independent mechanism (Eckford et al., 2012). Ivacaftor also potentiated channel activity of CFTR bearing other class III or IV mutants in Fisher rat thyroid cells (Yu et al., 2012; Van Goor et al., 2014) and rescued forskolin-induced swelling in rectal organoids bearing CFTR mutants with residual function (Dekkers et al., 2016a). A subsequent series of phase II and III clinical trials showed that ivacaftor reduced sweat chloride levels and improved FEV_1_ in CF patients bearing G551D (Accurso et al., 2010; Ramsey et al., 2011; Davies et al., 2013a,b; McKone et al., 2014) or one of other eight class III mutants (G178R, S549N, S549R, G551S, G1244E, S1251N, S1255P, and G1349D) (De Boeck et al., 2014) in at least one allele. For R117H, a residual function mutant, ivacaftor decreased sweat chloride levels of all patients, but only individuals older than 18 years and with a polythymidine tract variant of 5T presented improvements in FEV_1_ (Carter et al., 2015; Moss et al., 2015; Ronan et al., 2015). Currently, ivacaftor is available for CF patients bearing the aforementioned mutations.

Monotherapy with ivacaftor (Flume et al., 2012) or lumacaftor (Clancy et al., 2012) was unsuccessful in improving FEV_1_ of ΔF508-homozygous patients in phase II trials. A closer investigation of the pleiotropic defects caused by ΔF508 in CFTR reveals that combinations of drugs with different mechanism of actions will be required to obtain more efficient rescue of the mutated protein that may ultimately result in therapeutic levels in the patients (Okiyoneda et al., 2013; Phuan et al., 2014; Lopes-Pacheco et al., 2015; Veit et al., 2016a). Toward this goal, phase II and III trials tested the effects of co-administration of lumacaftor/ivacaftor. This approach reduced pulmonary exacerbations, slightly decreased sweat chloride levels and induced significant, but modest, improvements in FEV_1_ of ΔF508-homozygous patients (Boyle et al., 2014; Wainwright et al., 2015; Elborn et al., 2016). Recently, co-administration of lumacaftor/ivacaftor was licensed for ΔF508-homozygous patients.

Some flavonoids also increase channel open probability of CFTR mutants (Galietta et al., 2001; Pyle et al., 2010; Yu et al., 2011). In particular, genistein and curcumin have shown synergy with lumacaftor to enhance forskolin-induced swelling in rectal organoids bearing CFTR mutants ΔF508, G551D and S1251N (Dekkers et al., 2016c). Ivacaftor has shown clinical benefits when administered by itself, but its effects could be even greater when used with other compounds for CFTR bearing G551D or R117H (Gentzsch et al., 2016; Lin et al., 2016; Yu et al., 2016). In addition, new potentiators are under investigation to restore channel activity of CFTR mutants, including QBW251 (NCT02190604) and GLPG1837 (NCT02690519 and NCT02707562), as well as dual activity compounds that act as both correctors and potentiators, such as aminoarylthiazoles (Pedemonte et al., 2011; Pesce et al., 2015) and rattlesnake phospholipase A_2_ (Faure et al., 2016).

### Stabilizing the Protein at the Cell Surface

Stabilizers are agents that anchor CFTR channel at the PM and decrease protein degradation rate, thereby correcting the instability of class VI mutants. Low-temperature incubation of cells bearing ΔF508 rescue CFTR to the PM (rΔF508) (Sharma et al., 2001); however, the protein still presents reduced half-life due to both increased endocytosis (Swiatecka-Urban et al., 2005) and decreased recycling (Sharma et al., 2004). Lumacaftor also did not confer long-term stability of wt-CFTR for the mutant ΔF508 (He et al., 2013).

New treatments must rectify the intrinsic protein instability to rescue the steady state levels and augment CFTR residence time at the PM. In this line, hepatocyte growth factor (HGF) activated Rac1 signaling and promoted ΔF508 stabilization favoring interaction of CFTR and NHERF-1 (Moniz et al., 2013). Lumacaftor also promoted interaction of CFTR and NHERF-1 (Arora et al., 2014), but co-administration of HGF/lumacaftor further increased the rescue of ΔF508-CFTR and enhanced protein anchoring at the PM, compared to lumacaftor alone (Loureiro et al., 2015). Administration of VIP (Rafferty et al., 2009; Alshafie et al., 2014) or activation of EPAC1, an exchange protein directly activated by cAMP (Lobo et al., 2016), also stabilized CFTR at the PM by promoting its interaction with NHERF-1 and by decreasing its endocytosis rate.

In addition, inhibition of *S*-nitrosoglutathione reductase enhanced ΔF508 maturation and stability by preventing interaction of CFTR with Hsp70/Hsp90 organizing protein (Marozkina et al., 2010; Zaman et al., 2016). Cavosonstat (formerly N91115) increased S-nitrosoglutathione levels in pre-clinical studies and it is the first CFTR stabilizer in phase II trials being evaluated for ΔF508-homozygous patients using the combination lumacaftor/ivacaftor (NCT02589236) or for those patients bearing gating mutants and receiving ivacaftor (NCT02724527).

### Correcting the Splicing

Antisense oligonucleotides-based therapy is an emerging approach to correct class V mutations caused by alternative splicing that generate aberrant mRNA variants. About 11% of mutations-causing CF occur by incorrect splicing and this approach has shown to modulate the splicing and restore normal full-length CFTR transcript, as well as rescue functional CFTR protein (Bonini et al., 2015; Igreja et al., 2016).

A synthetic RNA oligonucleotide, QR-010, is being evaluated in two phase I trials to assess: (1) the effects of inhaled single and multiple doses in ΔF508-homozygous patients (NCT02532764), and (2) the effects of intranasal administration in ΔF508-homozygous and -heterozygous patients (NCT02564354).

## Precision Medicine: Breakthroughs and Challenges to Treating all CF Patients

From the first pathological description of the disease until the discovery of the CFTR gene and its correlation with CF, therapeutic interventions were only aimed at reducing clinical symptoms and end-organ complications. Nowadays, based on the accumulated knowledge about CFTR protein (synthesis, folding, trafficking and degradation), many studies have sought more specific treatments to restore expression, function and stability of CFTR mutants, thereby overcoming the primary molecular defect that causes CF. As such, CFTR modulators provided new perspectives and advances in the treatment of patients bearing common and rare CFTR mutations.

Many clinical trials have completed the tests of safety and efficacy of CFTR modulators (**Table 3**). The first breakthrough in personalizing CF treatments came with the approval of ivacaftor (Kalydeco^TM^ from Vertex Pharmaceuticals) for patients bearing G551D-CFTR in at least one allele, initially licensed by the FDA and later by the European Medicines Agency (EMA). Clinical studies showed that ivacaftor treatment improves FEV_1_ and almost normalizes sweat chloride levels (Accurso et al., 2010; Ramsey et al., 2011; Davies et al., 2013a,b), as well as reduces frequency of infections caused by *P. aeruginosa* (Rowe et al., 2014), enhances nutritional status (Borowitz et al., 2016), insulin secretion (Bellin et al., 2013) and quality of life (Quittner et al., 2015). Acute administration of ivacaftor also corrects smooth muscle abnormalities, improving airway distensibility and vascular tone (Adam et al., 2016). Clinical benefits obtained by ivacaftor treatment have proven durable effects, with no new safety concerns (Sawicki et al., 2015). Ivacaftor was later licensed for patients bearing one of the other eight gating mutations (De Boeck et al., 2014) and a mutant with residual function in at least one allele (Moss et al., 2015), increasing to 5–7% the number of individuals worldwide who may benefit from this pharmaceutical treatment. Despite these optimistic results, patients using ivacaftor should continue with conventional therapies, which have also been optimized, to prevent pulmonary exacerbations and complications (Smyth et al., 2014).

**Table 3:** Completed clinical trials of CFTR modulators in CF patients.

ClinicalTrials.gov ID (formerly name)	Phase	Subjects	Age (years)	Drug(s)	Follow up	Reference(s)
NCT00237380	2	Nonsense mutations^a,b^	≥18	Ataluren	56 days	Kerem et al., 2008
NCT00351078	2	Nonsense mutations^a,b^	≥18	Ataluren	112 days	Wilschanski et al., 2011
NCT00457821	2	G551D-homozygous and -heterozygous	≥18	Ivacaftor	28 days	Accurso et al., 2010, 2014
NCT00458341	2	Nonsense mutations^a,c,d^	6–18	Ataluren	28 days	Sermet-Gaudelus et al., 2010
NCT00803205	3	Nonsense mutations^a,c^	≥6	Ataluren	48 weeks	Kerem et al., 2014
NCT00865904	2	ΔF508-homozygous	≥18	Lumacaftor	28 days	Clancy et al., 2012
NCT00909532 (STRIVE)	3	G551D-homozygous and -heterozygous	≥12	Ivacaftor	48 weeks	Ramsey et al., 2011; Quittner et al., 2015
NCT00953706 (DISCOVER)	2	ΔF508-homozygous	≥12	Ivacaftor	16 weeks	Flume et al., 2012
NCT01117012 (PERSIST)	3	G551D-homozygous and -heterozygous	≥6	Ivacaftor	96 weeks	McKone et al., 2014
NCT01225211	2	ΔF508-homozygous and -heterozygous	≥18	Lumacaftor and Ivacaftor	56 days	Boyle et al., 2014
NCT01262352 (ENVISION)	2	G551D-homozygous and -heterozygous	≥6	Ivacaftor	48 weeks	Davies et al., 2013a,b
NCT01521338 (GOAL)	4	G551D-homozygous and -heterozygous	≥6	Ivacaftor	6 months	Rowe et al., 2014; O’Connor and Seegmiller, 2016
NCT01531673	2	ΔF508-homozygous and -heterozygous	≥12	VX-661 and/or Ivacaftor	28 days	^∗∗∗^
NCT01614457 (KONDUCT)	3	R117H-homozygous and -heterozygous	≥6	Ivacaftor	24 weeks	Moss et al., 2015
NCT01614470 (KONNECTION)	3	Non-G551D gating mutations in at least one allele^e^	≥6	Ivacaftor	24 weeks	De Boeck et al., 2014
NCT01685801	2	R117H and/or CFTR mutations with residual function in at least one allele ^b,f^, G551D and/or other gating mutations in at least one allele^e^	≥12	Ivacaftor	24 weeks	^∗∗∗^
NCT01705045 (KIWI)	3	G551D-homozygous and heterozygous	2–5	Ivacaftor	24 weeks	Davies et al., 2016
NCT01707290	3	Non-G551D gating or residual function mutations in at least one allele^b,e,f^	≥6	Ivacaftor	24 weeks	^∗∗∗^
NCT01807923 (TRAFFIC)	3	ΔF508-homozygous	≥12	Lumacaftor and Ivacaftor	24 weeks	Wainwright et al., 2015; Elborn et al., 2016
NCT01807949 (TRANSPORT)	3	ΔF508-homozygous	≥12	Lumacaftor and Ivacaftor	24 weeks	Wainwright et al., 2015; Elborn et al., 2016
NCT01897233	3	ΔF508-homozygous	6–11	Lumacaftor and Ivacaftor	24 weeks	^∗∗∗^
NCT01931839	3	ΔF508-homozygous	≥12	Lumacaftor and Ivacaftor	96 weeks	^∗∗∗^

*^a^G542X, W1282X; ^b^3849 + 10 kbC→T; ^C^R553X, R1162X; ^d^Q492X, E1104X, W846X, W882X, Q1313X; ^e^G178R, S549N, S549R, G551S, G970R, G1244E, S1251N, S1255P, G1349D; ^f^R117H, E56K, P67L, D110E, D110H, R117C, R347H, R352Q, A455E, D579G, S945L, L206W, R1070W, F1074L, D1152H, S1235R, D1270N, 2789 + 5G→A, 3272-26A→G, 711 + 5G→A, 3120G→A, 1811 + 1.6 kbA- > G, 711 + 3A→G, 1898 + 3A→G, 1898 + lG→A, 1717-lG→A, 1717-8G→A, 1342-2A→C, 405 + 3A→C, 1716G/A 1811 + lG→C, 1898 + 5G→T, 3850-3T→G, IVS14b + 5G→A, 1898 + lG→T, 4005 + 2T→C, 621 + 3A→G, 621 + lG→T. ^∗∗∗^Manuscript not available yet.*

Ataluren (Translarna^TM^ from PTC Therapeutics) was approved by the EMA for individuals who have Duchenne muscular dystrophy caused by nonsense mutation. After the inconclusive results from the first phase III trial, a second one is ongoing to evaluate the effects of ataluren in CF patients bearing nonsense mutations and not receiving inhaled aminoglycosides (NCT02139306).

Recently, the FDA and the EMA licensed the combination lumacaftor/ivacaftor (Orkambi^TM^ from Vertex Pharmaceuticals) for ΔF508-homozygous patients, adding a new pharmaceutical treatment to 40–45% of CF patients worldwide. Results from a phase II trial evaluating this combination in ΔF508-homozygous patients (Boyle et al., 2014) encouraged the pursuance of two long-term phase III trials (24 weeks) involving more than 1,100 people. Co-administration of lumacaftor/ivacaftor proved clinical effectiveness, despite fairly small improvement in FEV_1_ (Wainwright et al., 2015; Elborn et al., 2016). A longer-term (96 weeks) phase III trial showed that co-administration of lumacaftor/ivacaftor presents sustained benefit with decreased pulmonary exacerbations, reduced rate of lung function decline and improved nutritional status of ΔF508-homozygous patients (Konstan et al., 2016). Although it is still a glimmer of light at the end of the tunnel, this combination provides proof of concept that pleiotropic CFTR mutants can be rescued at therapeutic levels, representing a new hope to restore a healthy life to patients.

Intriguingly, studies using cell lines bearing ΔF508-CFTR showed that: (1) Chronic ivacaftor exposure (>1 μM) reduced CFTR correction obtained by lumacaftor treatment (Cholon et al., 2014; Veit et al., 2014); however, exposure to a lower concentration (<1 μM) of ivacaftor did not inhibit the functional correction obtained by lumacaftor (Matthes et al., 2016). Therefore, co-administration of lumacaftor/ivacaftor may present a dose-dependent inhibitory effect. Addition of C4 also reversed the negative effects of ivacaftor on lumacaftor-corrected CFTR (Bali et al., 2016b). (2) *P. aeruginosa* infection reduces CFTR chloride secretion stimulated by lumacaftor and/or ivacaftor treatments (Stanton et al., 2015). (3) In cells previously treated with lumacaftor, the PPQC system still recognizes the CFTR delivered at the PM as improperly folded and removes the protein from the cell surface (Loureiro et al., 2015). Taken together, the experimental results indeed may explain the modest findings in the clinical trials and highlight the importance of better evaluating drug-drug and drug-protein interactions for future drug combinations.

The introduction of Kalydeco^TM^ and Orkambi^TM^ to the market add feasible treatment for about 50% of all CF patients worldwide (**Table 4**). Unfortunately, there is still an unmet need for the other half of the CF population, who are ΔF508-heterozygous and bear a wide range of other variants (so-called orphan mutants). The patient registries and the clinical studies network have optimized the clinical phase of drug development due to heterogeneous distribution of CFTR variants (De Boeck et al., 2011; Rowe et al., 2012). Randomized controlled trials are suitable for common mutations, but many CFTR mutants are rare or even unique, requiring different approaches. As such, modified single-patient (‘N-of-1’) is an alternative trial design in which single patients serve as their own control by measuring outcome parameters after multiple cycles of on-off treatment (Zucker et al., 2010; Duan et al., 2013).

**Table 4:** Summary of Kalydeco^TM^ and Orkambi^TM^ approval for CF patients’ treatment.

Pharmaceutical treatment	CFTR mutations	Jurisdiction approved	Age group licensed
Kalydeco^TM^	G551D^∗^	United States, Europe and Canada	>2 years
		Australia and New Zealand	>6 years
	G178R, S549N, S549R, G551S, G1244E, S1251N, S1255P, G1349D^∗^	United States, Europe and Canada	>2 years
		Australia	>6 years
	R117H^∗^	United States	>2 years
		Europe and Canada	>18 years
Orkambi^TM^	ΔF508-homozygous	United States, Europe and Canada	>12 years

*^∗^Mutation in at least one allele.*

Rectal organoids have also shown to be a good model for identifying drug-responsive individuals with rare CFTR genotypes (Dekkers et al., 2016a; Vijftigschild et al., 2016). In addition, both Kalydeco^TM^ and Orkambi^TM^ are in ongoing clinical trials to: (1) evaluate the longer-term safety and efficacy of the treatments; (2) increase the panel of mutations that may benefit from these drugs; and (3) evaluate the safety and efficacy in younger patients. Since the progressive damage caused by CF starts during babyhood, earlier treatments are critical to offering the best chances of improving long-term outcomes.

In an attempt to achieve greater clinical outcomes, two new classes of CFTR modulators are under investigation in association with Orkambi^TM^: (1) PTI-428 is an amplifier that doubled the activity of the combination lumacaftor/ivacaftor in HBE cells bearing ΔF508-CFTR in both alleles (Proteostasis Therapeutics, 2016); and (2) cavosonstat is a stabilizer that improved ΔF508-CFTR stability at the PM after co-administration of lumacaftor/ivacaftor (Nivalis Therapeutics, 2016). Moreover, other correctors and potentiators are advancing in triple combination for treatment of ΔF508-homozygous and -heterozygous patients: GLPG1837/GLPG2222/GLPG2665 (Galapagos, 2015), VX-152/VX-661/ivacaftor and VX-440/VX-661/ivacaftor (Vertex Pharmaceuticals, 2015).

An important limitation that has to be addressed regarding these new pharmaceutical treatments is the high cost (over US$250,000 per patient per year), which renders difficult their acquisition for CF patients living in low-income countries. Investments in the identification of drugs already available and able to overcome the primary molecular defect of CF could be another way, cheaper and fast, to optimize treatments.

Soon after the CFTR gene was discovered and cloned (Kerem et al., 1989; Riordan et al., 1989), gene therapy was proposed to cure lung disease in CF. The idea of introducing a copy of wt-CFTR in airway cells to re-establish the production of functional protein seemed graceful and ‘simple’. Although CF is a ‘simple’ monogenic disorder, most approaches (viral and non-viral) failed to cross the trapped mucus, thereby resulting in inefficient transduction of CFTR gene, and some of them promoting immune response activation (Di Gioia et al., 2015; Quon and Rowe, 2016). Nevertheless, researchers have sought more efficient gene delivery systems. Poly (β-amino esters)-based biodegradable polymers were able to deliver a plasmid that encodes full-length CFTR in murine lungs and CFBE41o- cell lines expressing wt-CFTR or ΔF508 (Suk et al., 2014), as well as to penetrate in freshly expectorated mucus from CF patients (Mastorakos et al., 2015). Furthermore, repeated nebulization of pGM169/GL67A, a gene-liposome complex, showed feasible, safe and reproducible results in murine lungs (Alton et al., 2014), as well as stabilized lung function of CF patients, thereby presenting a significant, albeit modest, benefit in FEV_1_ in a phase II trial (NCT01621867) (Alton et al., 2015). These systems may also be an interesting approach to delivery of CFTR modulators, bringing some benefits in usefully exploited patients’ treatment: (1) non-invasive administration route by inhalation/nebulization; (2) delivering directly to a specific lung region or defined cell type; (3) reduced systemic side effects and no serum proteins sequestered; and (4) sustained release, which decrease times and dose of therapies to maintain the beneficial effects (Vij et al., 2010; Suk et al., 2014; Alton et al., 2015; Kim et al., 2015).

## CFTR Modulators Rescuing Mutants in Other ABC Transporters

Plasma membrane proteins belong to some of the largest families, encoding ion channels, transporters, aquaporins and ATP-powered pumps. Forty-nine proteins constitute the ABC transporters family in the human genome (Loo and Clarke, 2008; Vasiliou et al., 2009) and among these proteins, CFTR is unique in possessing a RD and in functioning as a chloride channel (Winter and Welsh, 1997; Gadsby et al., 2006; Serohijos et al., 2008). Mutations in ABC transporters have been linked to diseases and, since these transporters share a similar domain organization and structure, some CFTR modulators have been evaluated to rescue protein trafficking and function of other ABC transporters. Here are some examples: ABCA4 (Sabirzhanova et al., 2015), ABCB1 (or *P*-glycoprotein) (Wang et al., 2007b), ABCC6 (or multidrug resistance-associated protein 6) (Zhou et al., 2013), ABCC8 (or SUR1) (Sampson et al., 2013), and ABCG2 (Woodward et al., 2013). These findings show that CFTR modulators could be a promising treatment for several diseases caused by mutations in other ABC transporters.

## Conclusion

Since the discovery that CFTR loss-of-activity causes CF, the biological understanding of CFTR processing has advanced steadily and opened new perspectives for more sophisticated treatments, which act directly on the molecular defect that causes the disease. The presence of CFTR modulators in the market has affected positively the clinical outcomes of CF patients, representing a new dawn in their lives. Although there is still a long way to go in completely restoring a healthy life for all CF patients, research from the bench to the clinic is moving forward at an accelerated pace toward precision medicines, which would enable “the highest attainable standard of health,” as enshrined in the constitution of the World Health Organization.

## Author Contributions

The author confirms being the sole contributor of this work and approved it for publication.

## Conflict of Interest Statement

The author declares that the research was conducted in the absence of any commercial or financial relationships that could be construed as a potential conflict of interest.

## Footnotes

1 http://www.genet.sickkids.on.ca/Home.html

2 http://cftr2.org/

## Abbreviations

ABC: ATP-binding cassette
AHSA1: activator of 90 kDa Hsp ATPase homolog 1
ATP: adenosine triphosphate
cAMP: cyclic adenosine monophosphate
CAL: CFTR-associated ligand
CF: cystic fibrosis
CFBE: CF bronchial epithelial
CFTR: CF transmembrane conductance regulator
CHIP: carboxyl terminus of Hsc70-interacting protein
EPAC: exchange protein directly activated by cAMP
ER: endoplasmic reticulum
EMA: European Medicines Agency
ENaC: epithelial Na^+^ channel
ERAD: ER-associated degradation
FDA: Food and Drug Administration
FEV_1_: forced expiratory volume in 1 s
HBE: human bronchial epithelial
HDAC: histone deacetylase
HGF: hepatocyte growth factor
Hsc: heat shock cognate
Hsp: heat shock protein
HTS: high-throughput screening
NBD: nucleotide-binding domain
NHERF-1: Na^+^/H^+^ exchanger regulatory factor
PKA: protein kinase A
PKC: protein kinase C
PM: plasma membrane
PPQC: peripheral protein quality control
RD: regulatory domain
SNARE: soluble *N*-ethylmaleimide-sensitive factor attachment protein receptor
SUMO: small ubiquitin-like modifier
STX: syntaxin
TMD: transmembrane domain
VCP: vasolin-containing protein
VIP: vasoactive intestinal peptide
wt: wild type
